# Chronological Expression of *PITX2* and *SIX1* Genes and the Association between Their Polymorphisms and Chicken Meat Quality Traits

**DOI:** 10.3390/ani11020445

**Published:** 2021-02-08

**Authors:** Haiyue Cao, Wei Zhou, Yuge Tan, Xiuli Xu, Haiguang Mao, Xinyang Dong, Ningying Xu, Zhaozheng Yin

**Affiliations:** College of Animal Science, Zijingang Campus, Zhejiang University, Hangzhou 310058, China; 11817038@zju.edu.cn (H.C.); 21719002@zju.edu.cn (W.Z.); 21817066@zju.edu.cn (Y.T.); 21817083@zju.edu.cn (X.X.); maohaiguang@163.com (H.M.); sophiedxy@zju.edu.cn (X.D.); nyxu@zju.edu.cn (N.X.)

**Keywords:** *PITX2* gene, *SIX1* gene, chronological expression, polymorphisms, meat quality, chicken

## Abstract

**Simple Summary:**

The production of chickens plays an increasingly important role in people’s daily life because of the high nutritional quality and relatively low cost. Meanwhile, with the improvement of living standards, the requirements for meat quality have also increased significantly. Thus, the improvement of meat quality is becoming more and more important in chicken breeding. Studies have shown that meat quality is closely related to the development of skeletal muscle, which is a complex process regulated by many genes. Paired-like homeodomain transcription factor 2 (*PITX2*) and SIX homeobox 1 (*SIX1*) genes are involved in skeletal muscle development, and their polymorphisms were significantly associated with the carcass and body size traits of chickens in our previous studies. The objective of the present study was to detect the chronological expression of them on the pectoral muscles, and evaluate whether their single nucleotide polymorphisms (SNPs) are associated with the meat quality traits in chickens. Our results could provide useful information for the functional research of regulatory mechanisms of meat quality and marker assisted-selection for chicken production and quality.

**Abstract:**

Meat quality is closely related to the development of skeletal muscle, in which *PITX2* and *SIX1* genes play important regulatory roles. The present study firstly provided the data of chronological expression files of *PITX2* and *SIX1* genes in the post-hatching pectoral muscle and analyzed the association of their polymorphisms with the meat quality traits of Wuliang Mountain Black-bone (WLMB) chickens. The results showed that both *PITX2* and *SIX1* genes were weakly expressed in the second and third weeks, and then increased significantly from the third week to the fourth week. Furthermore, there was a significant positive correlation between the expression levels of the two genes. Twelve and one SNPs were detected in the chicken *PITX2* and *SIX1* genes, respectively, of which four SNPs (g.9830C > T, g.10073C > T, g.13335G > A, g.13726A > G) of the *PITX2* gene and one SNP (g.564G > A) of the *SIX1* gene were significantly associated with chicken meat quality traits. For the *PITX2* gene, chickens with the CT genotype of g.9830C > T showed the highest meat color L*, shear force (SF), pH, and the lowest electrical conductivity (EC), and drip loss (DL) (*p* < 0.05 or *p* < 0.01); chickens with the CC genotype of g.10073C > T had the lowest L*, pH, and the highest DL (*p* < 0.01). For the SIX1 gene, chickens with the GG genotype of g.564G > A had the highest (*p* < 0.05) SF and pH. Furthermore, pH had a significant correlation with all the other meat quality traits. The current study could contribute to the research of regulatory mechanisms of meat quality and lay the foundation for improving meat quality based on marker-assisted selection in chickens.

## 1. Introduction

Recently, more and more consumers demand high quality poultry production [1]. The decision of customers to purchase meat is partly influenced by meat quality parameters, such as tenderness, color, and juiciness [2]. Moreover, these meat quality parameters are of great significance in meat cutting and processing [3]. The recent development in the poultry meat industry and the increase in public demand for high-quality meat have brought about a challenge to breeders to increase the meat quality of poultry. With the development of molecular techniques, marker-assisted selection (MAS) provides the potential for accurate selection and accelerates the breeding process, such as the identification and utilization of single-nucleotide polymorphisms (SNPs), which are associated with these traits [4].

Meat quality is influenced by multiple genes and is closely related to the development of skeletal muscle composed of myofibers [5,6]. As important transcription factors, paired-like homeodomain transcription factor 2 (*PITX2*) and SIX homeobox 1 (*SIX1*) are implicated in myogenesis and skeletal muscle development [7,8,9]. In chicken, PITX2 acts with lymphoid enhancing factor-1 to regulate myogenic differentiation in somites and the proliferation of myogenic progenitor cells. Furthermore, within the limb bud, *PITX2* affects the number of myocytes/myofibers and thus the final muscle size [10]. During the development of chicken skeletal muscle, *SIX1* also plays a critical role in myoblast development and the transformation of muscle fibers [11]. The mutation of *PITX2* and the absence of *SIX1* could reduce myofiber number and size in mice [12,13]. Thus, *PITX2* and *SIX1* may affect meat quality traits.

Some studies have shown that the polymorphisms of *PITX2* and *SIX1* genes were associated with pig meat quality [14,15]. In our previous studies, the polymorphisms of *PITX2* and *SIX1* genes were significantly associated with chicken carcass and body size traits [16,17]. However, the association between their polymorphisms and meat quality traits of chicken has not been reported before, nor has the chronological expression profiles of them in the post-hatching pectoral muscle. Therefore, in the present study, the chronological expression profiles of *PITX2* and *SIX1* genes in the post-hatching pectoral muscle were detected, as well as the association between their polymorphisms and meat quality traits in Wuliang Mountain Black-Bone (WLMB) chickens. WLMB chicken is a Chinese indigenous breed that has good meat quality, feathered feet, green ear lobes, black bones, and a lower growth rate than the commercial broiler, which provides a valuable genetic resource for scientific research and further genetic improvement in the chicken industry [16]. The *PITX2* gene (Gene ID: 427549) of chicken is located on chromosomes 4 with 3 exons, and the *SIX1* gene (Gene ID: 693262) is located on chromosome 5 with 2 exons. The results of our study could provide useful information for the functional research of the *PITX2* and *SIX1* genes and marker assisted-selection for chicken production and quality.

## 2. Materials and Methods

All experiments in the present study were carried out in accordance with the Chinese Animal Welfare Guidelines and approved by the Animal Welfare Committee of the College of Animal Science of Zhejiang University (Approval Number: ZJU20190149).

### 2.1. Experimental Animals

A total of 408 300-day-old Wuliang Mountain Black-bone (WLMB) chickens were humanely slaughtered in two batches (200 chickens hatched in a different date from another 208 chickens) and measured for meat quality traits. At the same time, blood samples were taken from all 408 chickens and stored at −20 °C for genomic DNA extraction. According to the Standards of Agricultural Industry of the People’s Republic of China (NY/T 828-2004), the age of 300 days is a typical period to reflect the breed characteristics of poultry. Furthermore, at the age of the first week (1 W), second week (2 W), third week (3 W), fourth week (4 W), sixth week (6 W) and eighth week (8 W), 10 chickens were selected and humanely slaughtered at each week respectively (a total of 60 chickens) to analyze PITX2 and SIX1 genes expression in pectoral muscle. All chickens were female and randomly selected from Wuliang Mountain Black-bone Chicken Professional Cooperatives of Long Street (Jingdon, China), where they were reared in the same environment and conditions (the ambient temperature in the room was gradually reduced from 30 °C to 21 °C by 3 °C per week. The lighting program was as follows: 24 h light until 10 d of life and then 19 h light:5 h dark in the rest of the experiment. The animals had free access to water and feed for ad libitum consumption. The feed for chickens contained 18% CP and 2800 kcal of ME/kg).

### 2.2. Data Collection of Meat Quality Traits

After chickens were humanely slaughtered, the left side of the pectoral muscle was separated to measure for meat quality after 45 min post-mortem. The X-Rite Spectrophotometer model 968 (Grandville, MI, USA) was used to measure the meat color (L*: The lightness, a*: The red/green value, and b*: The yellow/blue value) of the pectoral muscle [18]. Shear force (SF) was measured using the shear machine (Digital Muscle Tenderometer of Model C-LM3, Northeast Agricultural University, Harbin, China). Electrical conductivity (EC) was measured by the LF-STAR apparatus (Matthäus, Nobitz, Germany). Meat pH was measured using the pH Star 6.05 machine (Matthäus, Nobitz, Germany). Three different points on the left side of the pectoral muscle were selected per sample to measure the meat color (L*, a*, b*), shear force, electrical conductivity, and pH [19]. The trimmed pectoral muscle samples were weighed (W_0_) and held at 4 °C for 24 h. Then the surface liquid was blotted using filter paper, and the pectoral muscle samples were weighed (W_1_) again. The drip loss (%) = (W_0_ − W_1_)/W_0_ × 100% [20].

### 2.3. DNA Extraction, PCR Amplification and Sequencing

Blood samples were collected and stored at −20 °C. Genomic DNA was extracted with the Genome DNA Extraction Kit (TIANGEN, Beijing, China) according to the guidance of the manufacturer’s instructions.

Primers [16,17] to amplify the exons of *PITX2* and *SIX1* genes are shown in Table 1. Since the second exon of the *SIX1* gene has a high GC content and POLY N (A, T, and C), amplification is extremely difficult. Thus, only the first exon of the *SIX1* gene was sequenced in the present study. The primer sequences were synthesized by Tsingke Biotech Co., Ltd. (Hangzhou, China).

A PCR instrument (Eppendorf, Hamburg Germany) was used to run the PCR amplification with a total volume of 50 μL mixture, which included 25 μL of 1-5TM 2 × High-Fidelity Master (Tsingke Biotech. Hangzhou, China), 2 μL of each primer (10 μmol/L), 2 μL genomic DNA and 21 μL double-distilled water (Sangon Biotech, Shanghai, China). The PCR cycling protocol was as follows: 98 °C for 5 min followed by 35 cycles of 98 °C for 10 s, 30 s at the annealing temperature (60 °C) and extension 72 °C for 40 s, ending with an extension cycle at 72 °C for 7 min. The PCR products were checked by 1% agarose gel electrophoresis. For each exon, a clear single band was obtained on agarose gel electrophoresis from PCR products, the length of which was consistent with the expected. Next, the PCR products were sequenced using the direct sequencing method by Tsingke Biotech Co., Ltd. (Hangzhou, China).

### 2.4. RNA Extraction, cDNA Synthesis and Quantitative Real-Time PCR (qPCR)

The separated pectoral muscles were frozen immediately in liquid nitrogen and stored at −80 °C. Total RNA was isolated from the pectoral muscles using TRIzol (Invitrogen, Carlsbad, CA, USA) according to the manufacturer’s instructions. The quality of the obtained RNA was checked by electrophoresis, and the amount and purity (OD260/OD280 ratio > 1.9) were measured by a NanoDropND2000 spectrophotometer (Thermo Fisher Scientific, Waltham, MA, USA). The cDNA was synthesized using the PrimeScript RT reagent Kit With gDNA Eraser (Takara, Dalian, China).

The primers for qPCR of chicken *PITX2* and *SIX1* genes were designed by the Primer Premier 5.0 software based on the sequences of chicken *PITX2* mRNA (NM_205010.1) and *SIX1* mRNA (NM_001044685). *β*-*actin* was used for the housekeeping gene to normalize the expression levels of them. The primers (Table 1) of chicken *PITX2*, *SIX1*, and *β*-*actin* mRNA [21] were synthesized by Tsingke Biotech Co., Ltd. (Hangzhou, China). An ABI StepOnePlus Real-Time PCR System (Applied Biosystems, Foster City, CA, USA) was used to perform the qPCR by using Takara SYBR Premix Ex Taq™ II kits (Takara, Dalian, China). A total volume of 20 μL PCR reaction mixture including 2 μL of cDNA, 0.8 μL of forward primer, 0.8 μL of reverse primer, 10 μL of 2 × SYBR Premix Ex Taq II, 0.4 μL of ROX Reference Dye (50 ×), and 6 μL of nuclease-free water. qPCR was performed in a 96-well optical plate (Applied Biosystems, Foster City, CA, USA) at 95 °C for 30 s, followed by 40 cycles of 95 °C for 5 s, and 60 °C for 30 s. Each sample was analyzed in triplicate, and the results were quantified using the 2^−ΔΔCT^ method.

### 2.5. Statistical Analysis

The Kolmogorov–Smirnov test of software SPSS v20.0 (IBM, Chicago, IL, USA) was used to check the normality of the expression data of *PITX2* and *SIX1* genes. Then, one-way ANOVA was used to analyze the expression of the *PITX2* and *SIX1* genes among different weeks in pectoral muscle. Multiple comparison results at different weeks were corrected by Bonferroni correction. We used SPSS v20.0 to calculate the Pearson correlation coefficients between the expression levels of the two genes (*PITX2* and *SIX1*) and the Pearson correlation coefficients between the meat quality traits.

The SeqMan II version 5.01 (DNAStar Inc., Madison, WI, USA) was used to analyze the result of the sequencing. Genotype frequency, allele frequency, polymorphism information content (*PIC*), heterozygosity (*He*), and the effective number of alleles (*Ne*) were calculated and the deviations from Hardy–Weinberg equilibrium (*HWE*) were checked.

The SNPs of three genotypes and more than 10 individuals in each genotype were used to perform association analysis with meat quality traits. The general linear model (GLM) was separately implemented on each SNP by SPSS 20.0. The linear model used was as follows:

*Y_ijk_* = *μ* + *G_i_* + *B_j_* + *e_ijk_* (*i* = 3, *j* = 2)

where $Y_{\mathrm{ijk}\;}$ = the phenotypic value of meat quality traits, μ = the overall mean, $G_{i}$ = effect of genotypes, $B_{j}$ = effect of birthdate, and $e_{\mathrm{ijk}}$ = random residual error. Multiple comparison results of the different genotypes were corrected by Bonferroni correction.

## 3. Results

### 3.1. The Chronological Expression of PITX2 and SIX1 Genes and Their Correlation

**Figure 1:**
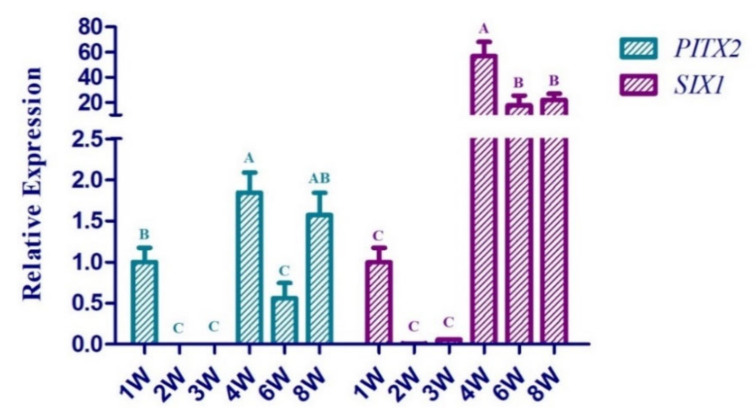
Relative mRNA expression levels of chicken *PITX2* and *SIX1* genes in the pectoral muscle at different weeks (n = 10 per week). Bars in the same group (*PITX2* or *SIX1* gene) with different capital letters indicate a highly significant difference (*p* < 0.01), and with the same capital letter indicate no significant difference (*p* < 0.05). Data are shown as the mean ± SD.

The chronological expression profiles of *PITX2* and *SIX1* genes in pectoral muscle are shown in Figure 1. Both the *PITX2* and *SIX1* genes were weakly expressed at 2 W and 3 W, and then increased significantly from 3 W to 4 W. The difference was that the expression levels of the *PITX2* and *SIX1* genes at 4W were 2- and 58-fold higher than those in the third week, respectively. From 4 W to 8 W, the expression trends of them were different. For the *PITX2* gene, the expression trend was firstly decreased and then increased. While for the *SIX1* gene, it was firstly decreased and then remained stable. Furthermore, there was an extremely significant correlation between the expression of *PITX2* and *SIX1* genes with *r* = 0.817.

### 3.2. Polymorphisms and Diversity Parameter of PITX2 and SIX1 Genes

In the present study, 12 SNPs (g.9830C > T and g.10073C > T in exon 1, g.12713C > T, g.12755C > T, g.12938G > A, g.12961C > T, g.13019G > A, g.13079G > A, g.13285G > A, g.13335G > A, g.13726A > G and g.13856C > T in exon 3) were detected in the *PITX2* gene, and one SNP (g.564C > T in exon) was detected in the *SIX1* gene. In our previous studies, all mutations of *PITX2* and *SIX1* genes were synonymous [16,17]. As shown in Table 2, except for g.13079G > A and g.13856C > T of the *PITX2* gene, all SNPs exhibited three genotypes. The genotypic distributions of all the SNPs were in the balance of *HWE* (*p* > 0.05) except two SNPs (g.12755C > T and g.13335G > A) of the *PITX2* gene and the SNP (g.564G > A) of the *SIX1* gene. Moreover, g.9830C > T, g.10073C > T, g.13335G > A, g.13726A > G of the *PITX2* gene, and g.564G > A of the *SIX1* gene were moderately polymorphic. In addition, g.9830C > T, g.10073C > T, g.13335G > A, g.13726A > G of the *PITX2* gene and g.564G > A of the *SIX1* gene were used to perform association analysis with meat quality traits.

### 3.3. Association of PITX2 and SIX1 Gene Polymorphisms with Meat Quality Traits

The results of the association analysis between the polymorphisms of the *PITX2* and *SIX1* genes and meat quality traits are shown in Table 3. Four SNPs of the *PITX2* gene were associated with meat quality traits. For the locus of g.9830C > T, compared with individuals with the CC genotype, individuals with the CT genotype had significantly higher (*p* < 0.05) meat color L* and significantly lower (*p* < 0.01) EC. Compared with individuals with the TT genotype, individuals with the CT genotype had significantly higher (*p* < 0.05) SF. In addition, individuals with the CT genotype had significantly higher (*p* < 0.01) pH and significantly lower (*p* < 0.01) DL than those with CC and TT genotypes. For the locus of g.10073C > T, the meat color L* of TT genotype individuals was significantly higher than that of CC (*p* < 0.01) and CT (*p* < 0.05) genotypes. Compared with CC genotype individuals, CT genotype individuals had significantly higher (*p* < 0.01) pH and significantly lower (*p* < 0.01) DL. For the locus of g.13335G > A, individuals with the GA genotype had a higher (*p* < 0.01) DL than those with GG and AA genotypes. For the locus of g.13726A > G, individuals with the AG genotype had significantly lower (*p* < 0.05) meat color a* than those with the AA genotype, and significantly lower (*p* < 0.01) EC than those with the GG genotype. Moreover, for the locus of g.13726A > G of the *SIX1* gene, individuals with the GG genotype had significantly higher (*p* < 0.05) SF than those with the AA genotype, and significantly higher (*p* < 0.01) pH than those with the GA and AA genotypes.

### 3.4. The Correlation among Meat Quality Traits

Pearson’s correlation coefficients among the meat quality traits are shown in Table 4. Besides the negative and significant (*p* < 0.01) correlation with EC and DL, pH was correlated (*p* < 0.01) with meat color (L*, a*, and b*) and SF positively, which indicated that pH had a significant correlation with all the other meat quality traits. Meat color L*, a*, and b* were highly positively correlated with each other (*p* < 0.01), and L* was also correlated (*p* < 0.01) with DL negatively. Besides the positive and significant (*p* < 0.01) correlation with a*, SF was correlated with EC (*p* < 0.01) and DL (*p* < 0.05) negatively.

## 4. Discussion

Meat quality can be used to evaluate meat suitability for fresh consumption and preservation for a specific period [23]. It is affected by a variety of factors, such as environment, nutritional level, and especially heredity, which could be considered as the major factor. Quite significant levels of heritability were obtained for meat pH, color and water-holding capacity in chickens and turkeys, demonstrating that selection should be useful to improve meat quality of poultry [24,25,26]. Compared with pigs, genetic studies on meat quality traits in poultry started later [3,27]. Thus, more research is needed to define the optimal breeding strategy for improving poultry meat quality. In the present study, we firstly detected the chronological expression profiles of *PITX2* and *SIX1* genes in the post-hatching pectoral muscle and evaluated whether their polymorphisms were associated with meat quality traits in WLMB chickens.

In birds, the number of skeletal muscle fibers is completely determined during the embryonic stage [5]. Thus, the growth of postnatal muscle mainly depends on the hypertrophy of muscle fibers, in which muscle satellite cells are necessary. After being activated, the muscle satellite cells proliferate and fuse with the muscle fibers, leading to hypertrophy [28]. In mouse, *PITX2* and *SIX1* genes arrest muscle satellite cell proliferation and promote differentiation to regulate the development of postnatal muscle [29,30,31]. Furthermore, *PITX2* can induce the high expression of *Pax3*, and the latter can induce the high expression of *SIX1* [32,33]. However, the regulatory effects of them on post-hatching chicken skeletal muscle have not been reported. Our study made a preliminary study on the chronological expression profile of *PITX2* and *SIX1* genes in the post-hatching pectoral muscle of chicken. The results showed that *PITX2* and *SIX1* genes played an important role in chicken skeletal muscle development after chicken hatching, in which they might play a synergistic role. Jinghai Yellow chicken, another Chinese indigenous chicken breed, grew very slowly until they were four weeks of age, after which the rate of growth increased gradually [34]. Combined with the results of the present study, it was reasonable to speculate that the 4 W was the turning point for skeletal muscles to enter the rapid development stage. However, whether there is an interaction between the two genes and how they interact also needs more in-depth research.

In the present study, 12 (g.9830C > T, g.10073C > T, g.12713C > T, g.12755C > T, g.12938G > A, g.12961C > T, g.13019G > A, g.13079G > A, g.13285G > A, g.13335G > A, g.13726A > G and g.13856C > T) and one SNPs (g.564C > T) were detected in the chicken *PITX2* and *SIX1* genes, respectively. After the association analysis, g.9830C > T, g.10073C > T, g.13335G > A, g.13726A > G of the *PITX2* gene, and g.564G > A of the *SIX1* gene were found to be significantly associated with chicken meat quality traits. For the *PITX2* gene, chickens with the CT genotype of g.9830C > T showed the highest meat color L*, shear force (SF), pH, and the lowest electrical conductivity (EC), drip loss (DL); chickens with the CC genotype of g.10073C > T had the lowest L*, pH, and the highest DL; chickens with the GA genotype of g.13335G > A had the highest DL; chickens with the AG genotype of g.13726A > G showed the lowest meat color a* and EC. For the *SIX1* gene, chickens with the GG genotype of g.564G > A had the highest SF and pH. Combined with our previous results, individual with the CT genotype of g.10073C > T at the *PITX2* gene had higher breast width, pH and lower drip loss; individuals with the GG genotype of g.13335G > C at *PITX2* gene had higher breast angle, shank circumference and lower drip loss; individuals with the GG genotype of g.564G > A at the *SIX1* gene had higher breast muscle weight, percentage of breast muscle and pH. Therefore, the CT genotype of g.10073C > T and the GG genotype of g.13335G > C at *PITX2* may serve as molecular markers to improve both body size and meat quality traits of WLMB chickens; and the GG genotype of g.564G > A at the *SIX1* gene may serve as a molecular marker to improve both carcass and meat quality traits [16,17].

In this study, g.9830C > T and g.10073C > T of *PITX2* and g.564G > A of *SIX1* were significantly associated with pH, which had a significant correlation with all the other meat quality traits indicating that pH was a key point for chicken meat quality [3,35]. The pH of chicken pectoral muscle is strongly related to muscle glycolysis [27,35]. Coincidentally, *PITX2* and *SIX1* could regulate the glycolytic rate of mouse muscle and human melanoma, respectively [12,36]. Thus, we wonder whether the *PITX2* and *SIX1* genes could adjust the pH by influencing the muscle glycolysis rate to further affect the meat quality of chicken. It is obvious that more in-depth functional study of how *PITX2* and *SIX1* genes regulate the meat quality of chickens is still needed.

## 5. Conclusions

In summary, the present study firstly provided the data of chronological expression files of *PITX2* and *SIX1* genes in the post-hatching pectoral muscle, and analyzed the association of their polymorphisms with the meat quality traits of chickens. The results indicated that the *PITX2* and *SIX1* genes might play important roles in the development of post-hatching pectoral muscle of chicken, and their polymorphisms were significantly associated with the meat quality traits of WLMB chickens, which could contribute to the research of regulatory mechanisms of meat quality and lay the foundation for improving meat quality based on marker-assisted selection in chickens.

## Figures and Tables

**Table 1:** Primer sequences for amplifying the exons and qPCR of the chicken *PITX2* and *SIX1* gene.

Primer ^1^	Sequence (5′~3′)	Product Length (bp)
*PITX2*-1	F: GGGCACACGCGCTCCTT	430
	R: CTCGCCCTCTACAACCGAT	
*PITX2*-2	F: AGCGGTAACGGACAGCAAC	717
	R: GCCAATGGTTTCCGTAGC	
*PITX2*-3	F: CAGCGTTCTTCCCTGTGGT	1538
	R: CCGAAAAAGTGCGGCGTT	
*SIX1*-1	F: CGCAGCCTCAGCCTCAGC	1185
	R: AAGGCACCGAACAAAGGC	
*PITX2*-m	F: CGTCCTCTCGCCGATGAGTTGC	132
	R: GCGGGCTGCTGGTGATGG	
*SIX1*-m	F: AGGGAGAACACGGAGAACAAC	112
	R: GCGGGGGTGAAAATTCTTCCT	
*β*-*actin*-m	F: ACGTCGCACTGGATTTCGAG	282
	R: TGTCAGCAATGCCAGGGTAC	

^1^* PITX2*-1, *PITX2*-2, *PITX2*-3, and *SIX1*-1: the primer pairs of chicken *PITX2* gene’s exon 1, exon 2, exon 3, and *SIX1* gene’s exon 1 in PCR amplification, respectively; *PITX2*-m, *SIX1*-m, and *β*-*actin*-m: The primer pairs of chicken *PITX2*, *SIX1*, and *β*-*actin* mRNA in qPCR, respectively.

**Table 2:** Genotype frequency, allele frequency, and diversity parameter in the *PITX2* and *SIX1* genes in Wuliang Mountain Black-bone chickens.

Gene	SNPs ^1^	Genotypic Frequency			Allelic Frequency		*p*-Value ^2^	*PIC* ^3^	*He* ^4^	*Ne* ^5^
*PITX2*		CC	CT	TT	C	T				
	g.9830C > T	0.65 (265)	0.31 (127)	0.04 (16)	0.81 (657)	0.19 (159)	0.872	0.265	0.314	1.457
	g.10073C > T	0.58 (237)	0.38 (153)	0.04 (18)	0.77 (627)	0.23 (189)	0.279	0.293	0.356	1.553
	g.12713C > T	0.86 (352)	0.13 (52)	0.01 (4)	0.93 (756)	0.07 (60)	0.192	0.127	0.136	1.158
	g.12755C > T	0.90 (366)	0.09 (36)	0.01 (6)	0.94 (768)	0.06 (48)	0.000	0.105	0.111	1.125
		GG	GA	AA	G	A				
	g.12938G > A	0.94 (385)	0.05 (22)	0.00 (1)	0.97 (792)	0.03 (24)	0.262	0.055	0.057	1.061
		CC	CT	TT	C	T				
	g.12961C > T	0.82 (336)	0.16 (67)	0.01 (5)	0.91 (739)	0.09 (77)	0.428	0.156	0.171	1.206
		GG	GA	AA	G	A				
	g.13019G > A	0.88 (357)	0.12 (47)	0.01 (4)	0.93 (761)	0.07 (55)	0.091	0.118	0.126	1.144
	g.13079G > A	0.96 (393)	0.04 (15)	0.00 (0)	0.98 (801)	0.02 (15)	0.705	0.035	0.036	1.037
	g.13285G > A	0.84 (342)	0.15 (61)	0.01 (5)	0.91 (745)	0.09 (71)	0.234	0.146	0.159	1.189
	g.13335G > A	0.50 (203)	0.32 (129)	0.19 (76)	0.66 (535)	0.34 (281)	0.000	0.350	0.452	1.823
		AA	AG	GG	A	G				
	g.13726A > G	0.37 (151)	0.47 (192)	0.16 (65)	0.61 (494)	0.39 (322)	0.761	0.364	0.478	1.915
		CC	CT	TT	C	T				
	g.13856C > T	0.99 (406)	0.00 (2)	0.00 (0)	1.00 (814)	0.00 (2)	0.960	0.005	0.005	1.005
*SIX1*		GG	GA	AA	G	A				
	g.564G > A	0.73 (299)	0.23 (94)	0.04 (15)	0.85 (692)	0.15 (124)	0.032	0.225	0.258	1.347

^1^ SNPs: Single nucleotide polymorphism sites. ^2^ *p*-Value: The *χ*^2^ test of Hardy–Weinberg equilibrium, *p* > 0.05 suggests the population conforms to Hardy–Weinberg equilibrium. ^3^ *PIC*: Polymorphism information content. *PIC* > 0.5 means highly polymorphic, 0.25 < *PIC* < 0.5 signifies moderate polymorphism, *PIC* < 0.25 shows low polymorphism [22]. ^4^ *He*: Heterozygosity. ^5^ *Ne*: Effective number of alleles.

**Table 3:** Association of SNPs of *PITX2* and *SIX1* gene with meat quality traits in Wulang Mountain Black-bone chickens (MEAN ± SE).

Gene	SNPs	Genotypes	L*	a*	b*	SF (N)	EC (mS/cm)	pH	DL (%)
*PITX2*	g.9830C > T	CC (265)	44.95 ± 0.35 ^b^	5.28 ± 0.14	9.17 ± 0.21	29.20 ± 0.73 ^ab^	4.71 ± 0.27 ^A^	5.97 ± 0.07 ^B^	3.13 ± 0.12 ^A^
		CT (127)	46.51 ± 0.50 ^a^	5.37 ± 0.16	9.55 ± 0.24	31.24 ± 1.04 ^a^	3.04 ± 0.23 ^B^	6.48 ± 0.05 ^A^	2.20 ± 0.16 ^B^
		TT (16)	44.47 ± 1.70 ^ab^	4.38 ± 0.45	9.13 ± 0.89	22.82 ± 2.48 ^b^	4.57 ± 0.47 ^AB^	5.38 ± 0.38 ^B^	3.56 ± 0.44 ^A^
	g.10073C > T	CC (237)	45.02 ± 0.37 ^B^	5.32 ± 0.15	9.23 ± 0.22	29.14 ± 0.78	4.59 ± 0.27	5.97 ± 0.07 ^B^	3.23 ± 0.13 ^A^
		CT (153)	45.70 ± 0.44 ^AB^	5.28 ± 0.15	9.35 ± 0.22	30.10 ± 0.89	3.79 ± 0.39	6.31 ± 0.07 ^A^	2.41 ± 0.14 ^B^
		TT (18)	48.79 ± 1.23 ^A^	5.31 ± 0.32	9.82 ± 0.65	29.28 ± 2.83	4.65 ± 0.71	6.27 ± 0.20 ^AB^	2.44 ± 0.30 ^AB^
	g.13335G > A	GG (203)	45.71 ± 0.39	5.37 ± 0.14	9.41 ± 0.21	30.66 ± 0.84	4.45 ± 0.35	6.17 ± 0.07	2.81 ± 0.13 ^B^
		GA (129)	44.64 ± 0.49	5.27 ± 0.20	9.37 ± 0.31	28.18 ± 1.01	3.98 ± 0.26	5.99 ± 0.09	3.27 ± 0.18 ^A^
		AA (76)	45.91 ± 0.72	5.33 ± 0.25	9.22 ± 0.38	28.89 ± 1.42	5.40 ± 0.74	6.15 ± 0.12	2.47 ± 0.18 ^B^
	g.13726A > G	AA (151)	45.78 ± 0.51	5.64 ± 0.21 ^a^	9.76 ± 0.29	29.43 ± 1.02	4.57 ± 0.43 ^AB^	6.15 ± 0.08	2.84 ± 0.17
		AG (192)	44.90 ± 0.39	5.02 ± 0.14 ^b^	9.01 ± 0.22	29.67 ± 0.86	3.70 ± 0.18 ^B^	6.11 ± 0.07	2.94 ± 0.14
		GG (65)	46.02 ± 0.68	5.46 ± 0.26 ^ab^	9.54 ± 0.42	29.22 ± 1.38	5.70 ± 0.22 ^A^	5.92 ± 0.14	3.04 ± 0.23
*SIX1*	g.564G > A	GG (299)	45.79 ± 0.33	5.44 ± 0.12	9.44 ± 0.18	29.85 ± 0.67 ^a^	4.31 ± 0.25	6.21 ± 0.05 ^a^	2.82 ± 0.11
		GA (94)	45.28 ± 0.56	5.17 ± 0.20	9.45 ± 0.35	29.23 ± 1.15 ^ab^	4.59 ± 0.50	5.94 ± 0.11 ^b^	2.90 ± 0.18
		AA (15)	43.05 ± 1.10	4.72 ± 0.63	7.89 ± 0.70	22.63 ± 2.31 ^b^	4.22 ± 0.72	5.59 ± 0.35 ^b^	3.28 ± 0.39

L*, a* and b*: Meat color L*, a* and b* define the lightness, red/green and yellow/blue values, respectively [18]. SF: Shear force. EC: Electrical conductivity. DL: Drip loss. Means with different lowercase superscripts differ significantly (*p* < 0.05); means with different capital superscripts differ highly significantly (*p* < 0.01); means with the same letter do not differ significantly (*p* > 0.05).

**Table 4:** Correlation coefficients (r) among the meat quality traits of Wuliang Mountain Black-bone chickens.

Traits	L*	a*	b*	SF	EC	pH	DL
L*	1						
a*	0.43 **	1					
b*	0.54 **	0.76 **	1				
SF	0.03	0.13 **	0.05	1			
EC	0.07	0.01	−0.10	−0.22 **	1		
pH	0.22 **	0.16 **	0.13 **	0.39 **	−0.38 **	1	
DL	−0.24 **	−0.02	−0.03	−0.10 *	0.07	−0.35 **	1

L*, a* and b*: Meat color L*, a* and b* define the lightness, red/green and yellow/blue values, respectively [18]. SF: Shear force.EC: Electrical conductivity. DL: Drip loss. ** indicates a significant correlation at the level of 0.01 (2-tailed), and * indicates a significant correlation at the level of 0.05 (2-tailed).

## Data Availability

The data presented in this study are available on request from the corresponding author. The data are not publicly available due to privacy.
